# Open-source, 3D-printed Peristaltic Pumps for Small Volume Point-of-Care Liquid Handling

**DOI:** 10.1038/s41598-020-58246-6

**Published:** 2020-01-31

**Authors:** Michael R. Behrens, Haley C. Fuller, Emily R. Swist, Jingwen Wu, Md. Mydul Islam, Zhicheng Long, Warren C. Ruder, Robert Steward

**Affiliations:** 1 Department of Bioengineering, University of Pittsburgh, Pittsburgh, PA United States; 2 Department of Mechanical and Aerospace Engineering, University of Central Florida, Orlando, FL United States

**Keywords:** Lab-on-a-chip, Biomedical engineering

## Abstract

Microfluidic technologies are frequently employed as point-of-care diagnostic tools for improving time-to-diagnosis and improving patient outcomes in clinical settings. These microfluidic devices often are designed to operate with peripheral equipment for liquid handling that increases the cost and complexity of these systems and reduces their potential for widespread adoption in low resource healthcare applications. Here, we present a low-cost (~$120), open-source peristaltic pump constructed with a combination of three dimensional (3D)-printed parts and common hardware, which is amenable to deployment with microfluidic devices for point-of-care diagnostics. This pump accepts commonly available silicone rubber tubing in a range of sizes from 1.5 to 3 mm, and is capable of producing flow rates up to 1.6 mL min^−1^. This device is programmed with an Arduino microcontroller, allowing for custom flow profiles to fit a wide range of low volume liquid handling applications including precision liquid aliquoting, flow control within microfluidics, and generation of physiologically relevant forces for studying cellular mechanobiology within microfluidic systems.

## Introduction

Microfluidic systems are ubiquitous tools within science and engineering laboratories around the world that enable low-cost and high throughput analysis via the miniaturization and parallelization of experimental systems. To enable broader applications and lower the barrier to entry for using microfluidic technology, developing open-source and low-cost tools for handling fluids is a promising avenue of research^1–3^. Open-source microfluidic tools could be particularly impactful when used for point-of-care diagnostics in resource limited settings. The growing use of microfluidics in point-of-care diagnostic roles is driven by the desire for more personalized medical treatments that are tailored to the specific pathologies identified in the patient^4–7^. This is because assays that can be performed at the point of care dramatically improve time-to-diagnosis, leading to improvements in medical practitioner decision making and patient outcomes^8^. In order for point-of-care diagnostic devices to be widely adopted into more clinical settings so that they can effectively improve healthcare outcomes, advances must be made in key enabling technologies, including improved microfluidic device designs, and improved peripheral systems for fluid actuation and sensing^9^. Increasing the use of open-source tools for future point-of-care diagnostic tools would enable reductions in system cost, and increase ease of use.

**Figure 1:**
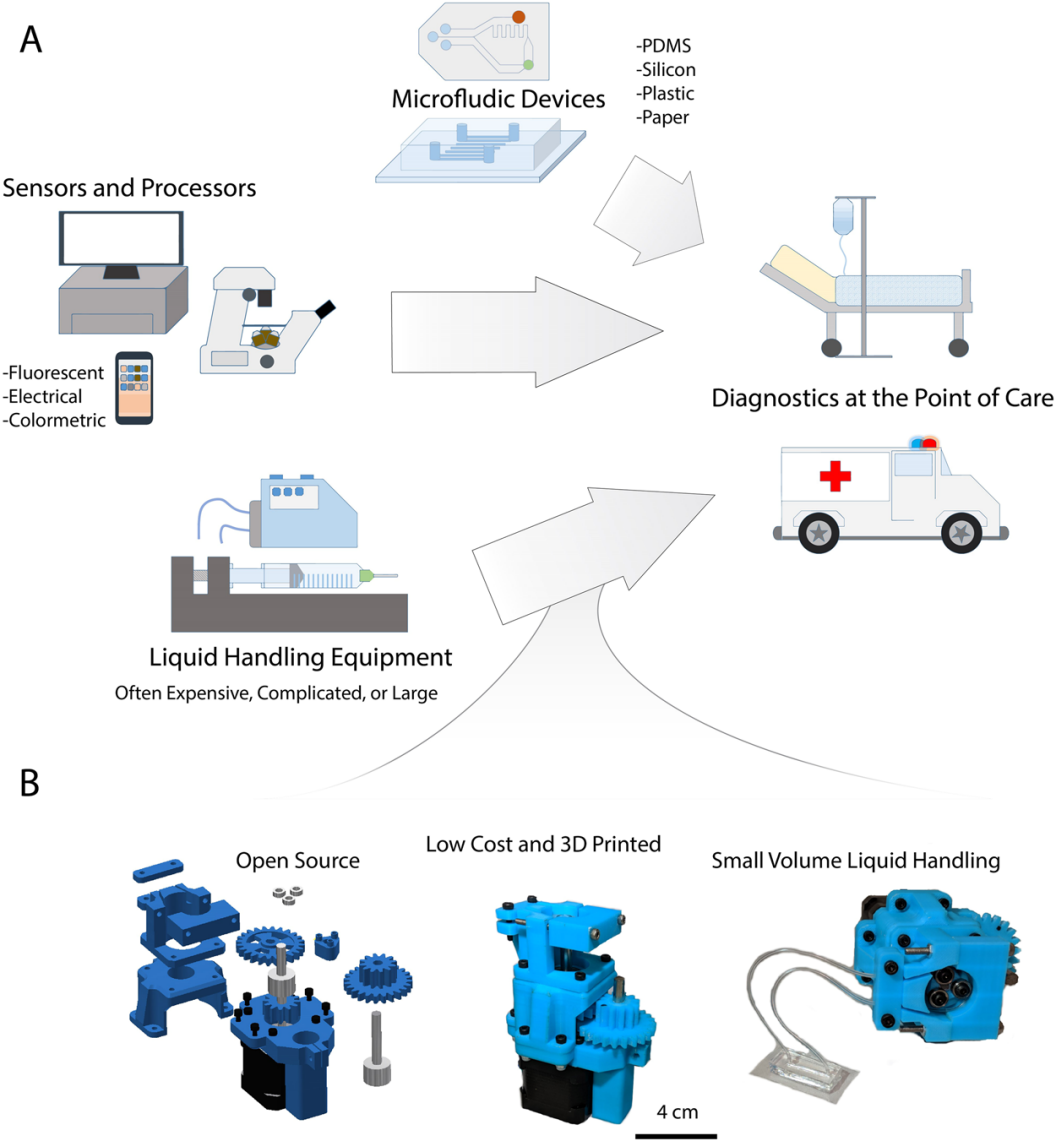
Liquid Handling for Point-of-Care Diagnostics. (**A**) Diagnostic devices for point-of-care applications often require integration of microfluidics with complicated and expensive peripheral equipment for signal readout and for liquid handling. (**B**) As a low-cost alternative to complex and expensive liquid handling equipment, we have developed an open-source, 3D-printed programmable peristaltic pump that can be used for precision low volume liquid handling and deployed with point-of-care diagnostic tools. The pump is assembled with a combination of 3D-printed parts and commonly available hardware, and is programmable via an Arduino microcontroller.

Microfluidic point-of-care diagnostic systems must include three basic components: the physical microfluidic device, systems to read the assay output, and systems to control the flow of liquid reagents (Fig. 1A). The physical microfluidic device can be manufactured from a wide range of low-cost materials, including PDMS^10^, glass^11^, plastic^12^, and paper^13–15^, and commonly takes the form of a single use disposable cassette^4^. To read the output from the assay, sometimes expensive computer systems, sensors and microscopes are used, while some assays are designed to be read out by visual inspection^15^.

Most microfluidic systems require precisely controlled fluid flow. While there are microfluidic systems that employ sophisticated on-chip pumps for liquid handling^16–20^, these devices are often complex to manufacture, expensive, or limited to low flow rates, which may limit their potential for mass adoption in point-of-care diagnostic systems. Instead, most microfluidic devices employ off-chip pumps to provide pressure and flow rate control. Many innovative off-chip pumps have been developed for microfluidics, including simple, ultra-low cost (<$10) reinforced latex balloons inflated to provide a steady pressure source^21^, more expensive (<$1000), yet programmable, low volume piezoelectric pumps^22^, and costly (>$1000), but sophisticated and robust syringe pumps^22,23^. Syringe pumps, in particular, are among the most common type of pump used for laboratory microfluidics. Syringe pumps can be set up to deliver constant flow rates, or constant pressure if feedback control is employed^1^. However, syringe pumps are not without disadvantages for many applications. Syringe pumps are limited to dispensing fixed volumes of liquid limited by the syringe volume, and they are not capable of driving recirculating flow within a closed system. Additionally, common syringe pumps used in scientific laboratories, such those produced by companies like Harvard Apparatus, can be prohibitively expensive for adoption in low resource clinical settings. Peristaltic pumps provide an alternative to syringe pumps which are better suited for some applications. These devices pump liquid by cyclically squeezing a flexible tube against a rigid housing^24^. Unlike syringe pumps they can be used drive recirculating flow in a closed fluidic circuit, and they are physically isolated from the sample by the walls of the tubing, which reduces contamination and safety concerns. Additionally, peristaltic pumps are well suited for dosing and metering fluids^25^, tasks for which they are widely employed for large volume liquid handling applications. Peristaltic pumps are employed in many microfluidic applications^26–28^, particularly those requiring recirculating flow for cell culture^27^. Several classes of on-chip integrated microfluidic peristaltic pumps have been developed, in which channels within a PDMS microfluidic device are squeezed by external hardware such as rollers^16^, magnets^19,20^, or by adjacent pressurized microfluidic channels^10,29^. While integrated pumps allow more precise control of small liquid volumes, integrated pump designs constrain the geometry of the microfluidic device. Additionally, integrated pumps often require complex fabrication procedures when compared to microfluidic devices powered by off-chip peristaltic pumps. However, commercial, off-chip peristaltic pumps designed for microscale liquid handling, similar to commercial syringe pumps, can be prohibitively expensive for many point-of-care diagnostic systems.

Here, we present the design of a low-cost (~$120) open-source, 3D-printed peristaltic pump that can be manufactured using common tools and hardware, that is designed for microliter-scale liquid handling and amenable to deployment with diagnostic microfluidic systems (Fig. 1B). This pump operates by peristaltic action of rolling ball bearings applying pressure to the outside of small diameter tubing. It is modular, able to accept a range of tubing diameters by quickly swapping out 3D-printed parts. The pump is driven by a stepper motor and power is transmitted to the pump through a 3D-printed 4:1 gearbox to increase the available torque. The motor is programmable via an open-source microcontroller (Arduino) to precisely control flow rate and direction. Here, we characterize the performance of this pump and demonstrate its applicability for a variety of small volume precision liquid handling applications.

## Results

### Pump design

**Figure 2:**
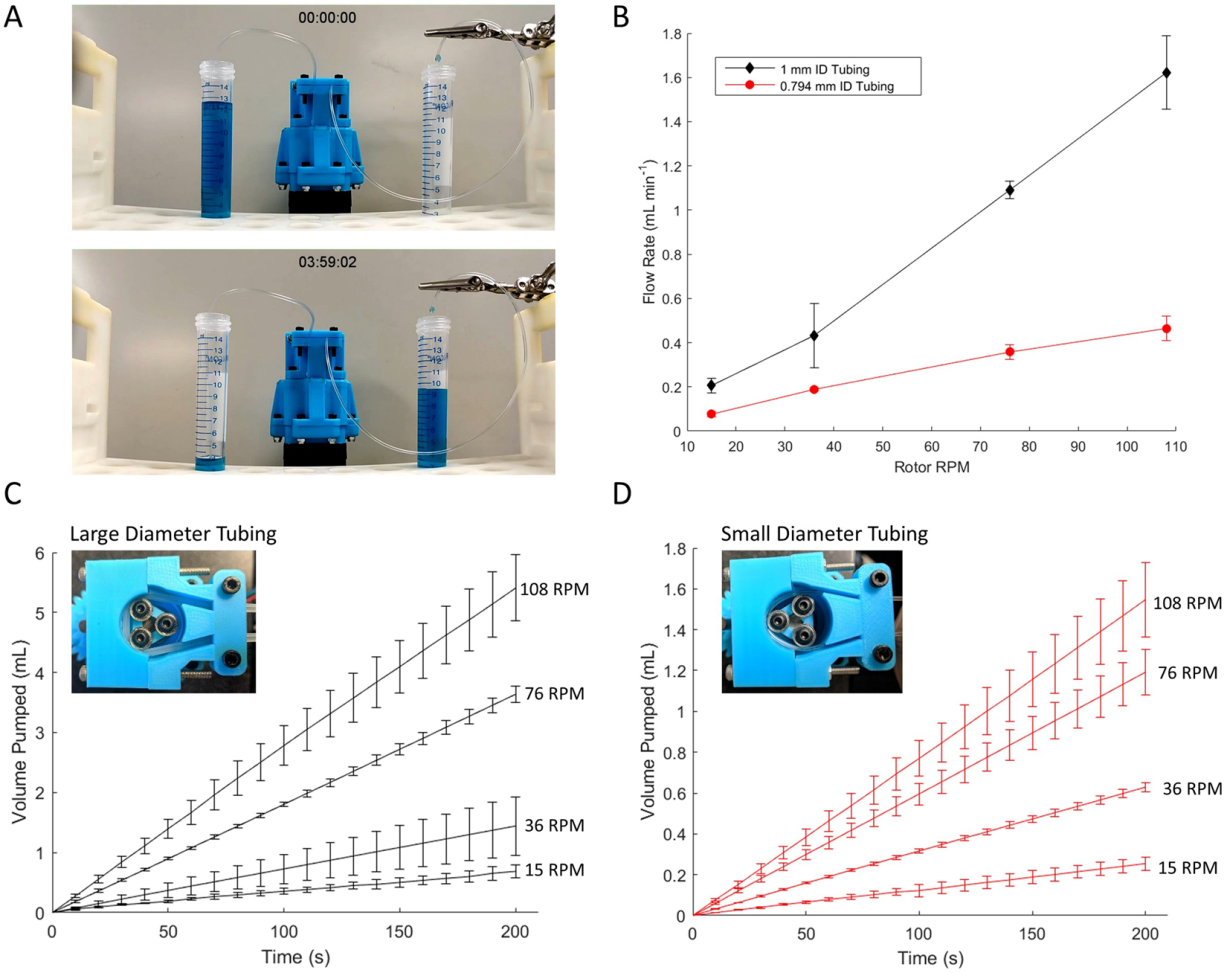
Flow Rate. (**A**) The pump uses peristaltic motion of rotating ball bearings with silicone tubing to transfer fluid. (**B**) Flow rate is controlled by varying the rotation speed of the pump, or by varying the diameter of the tubing. (**C**) Total volume pumped over time with 3 mm OD, 1 mm ID tubing, at four different pump rotation speeds. (**D**) Total volume pumped over time with 1.59 mm OD, 0.79 mm ID tubing. Traces represent the average results for three independent trials, with error bars representing one standard deviation above and below the mean.

The system architecture of the pump is subdivided into two basic subsystems: the electrical system, containing the power supply, microcontroller and motor driver, and the mechanical pump, based on a stepper motor and acrylonitrile butadiene styrene (ABS) 3D-printed components, and assembled with basic hardware. A complete bill of materials and assembly instruction set is provided in the supplementary materials (Supplementary Fig. S1, Supplementary Slide Deck). An Arduino microcontroller acts as the onboard central processing unit of the pump, and can be programmed using the free Arduino integrated development environment (IDE) via a USB connection to a personal computer. The microcontroller is responsible for sending step and direction commands to a EasyDriver stepper motor driver, which controls the rotation speed and direction of the pump rotor. Power is transmitted to the pump rotor via a 3D-printed 4:1 gearbox that is mounted to the stepper motor. The pump mounted on top of the gearbox is composed of two key components: the rotor and the stator. The rotor body is 3D-printed, and three ball bearings are secured to the rotor in a triangular configuration. These form the rollers which provide force on the tubing during pumping. The stator is composed of three 3D-printed pieces: a base which is specific to the tubing diameter being used, and two clamps that secure the tubing in place. The tubing is clamped between the rotor and the stator, so that when the rotor rotates the ball bearings squeeze the tubing against the stator. Peristalsis is achieved by cyclical compression of the tubing by the rotor, which provides force which drives fluid through the tubing (Fig. 2A).

### Flow rate

The rate of fluid flow through the pump is controlled by angular velocity of the rotor, which is encoded into the commands delivered to the motor controller by the Arduino, and by the diameter of tubing used. The pump was tested for two different sizes of silicone tubing: 1.59 mm (0.0625”) outer diameter (OD), 0.79 mm (0.03125”) inner diameter (ID) tubing, and 3 mm OD/1 mm ID tubing. Each tube had a durometer hardness rating of 50 A, designed to be used with peristaltic pumps. To switch between the two sizes of tubing, two different sizes of stator were designed and 3D-printed, and can be quickly swapped out to modulate the pump to accommodate different tube diameters.

To test the pump rate as a function of rotation speed, the pump was set up to pump deionized water onto a precision analytical balance. The balance was programmed to record real time weight measurements once per second. The pump rotation speed was determined by counting the number of rotations over a period of 2 min. Results of this experiment show that the pump rate is dependent on both the tubing diameter, and on the rotation speed of the pump (Fig. 2B). For a given rotation speed and tubing size, the pump maintains a constant average pump rate, resulting in a linear increase in pumped fluid volume over time (Fig. 2C,D). Friction constraints arising from manufacturing tolerances limit the practical rotational speed to approximately 108 revolutions per minute (RPM), at which speed the pump achieves 0.46 mL min^−1^ with the 1.59 mm tubing, and 1.62 mL min^−1^ with the 3 mm tubing. Variation in pump rate can be observed when tubing is clamped into the stator with differing levels of tension applied to the tubing, which accounts for the majority of variation across trials. Once tubing is securely clamped in place, the pump rate is highly uniform (Supplementary Fig. S2). Furthermore, since the hydrodynamic pressure drop in microfluidic channels is generally very large, we characterized the flow rate vs pressure drop generated by the system, and also calculated the power consumption and efficiency of our system. These performance benchmarks are depicted in Supplementary Figs S5 and S6.

### Pressure generation for microfluidics

**Figure 3:**
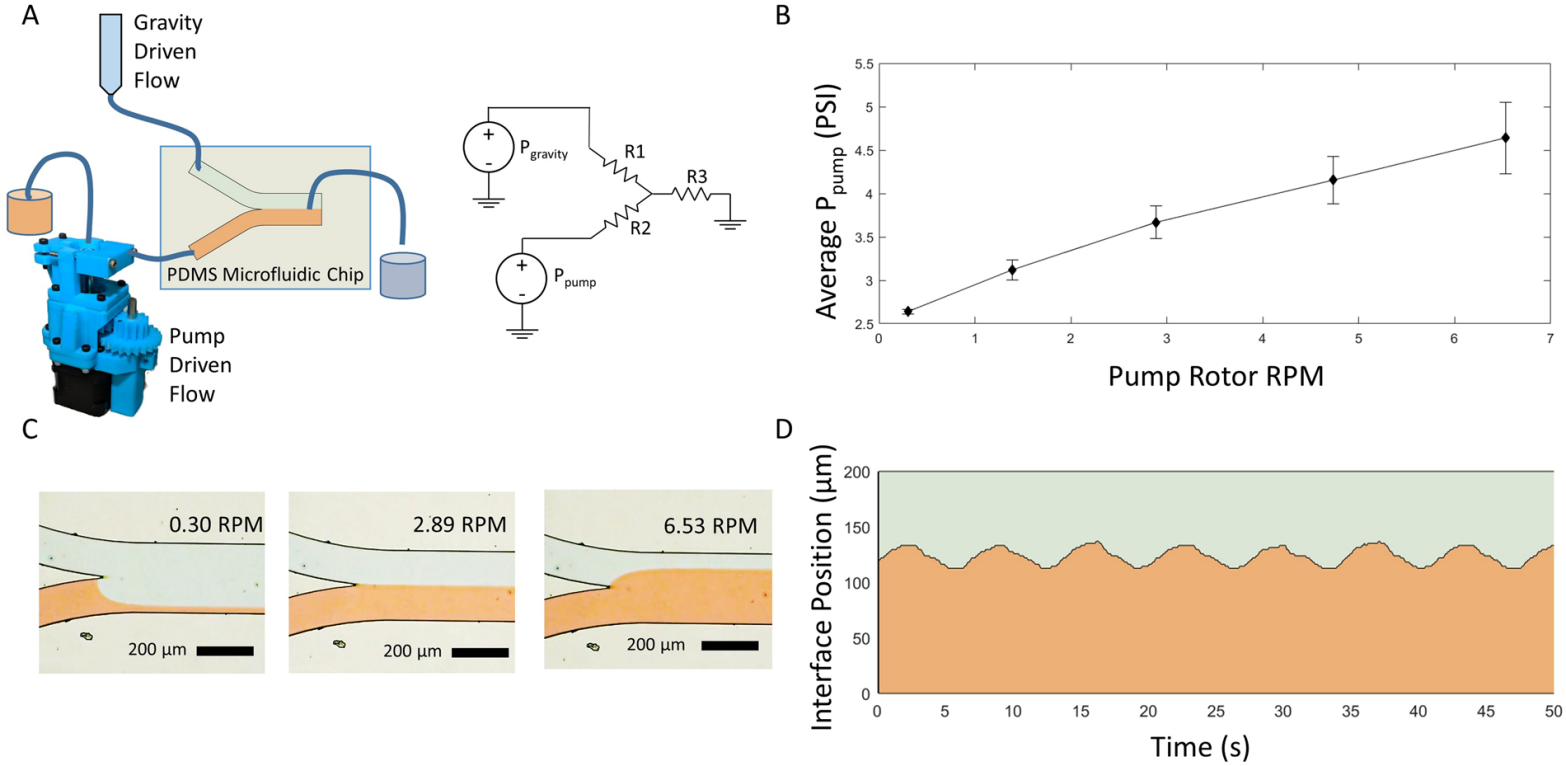
Pressure driven flow for microfluidics. (**A**) The peristaltic pump was set up to pump water supplemented with red food coloring through a PDMS microfluidic device with a Y-channel configuration. A syringe with water and blue food coloring was attached to the other inlet of the Y channel, and suspended 2.47 m above the microfluidic device to create a constant pressure source. Using an electrical circuit analysis analogy for microfluidics, the unknown pressure generated by the pump was calculated by observing the position of a laminar flow interface, and the known pressure from the gravity driven flow. (**B**) Pressure supplied by the pump is a function of rotor RPM. Data represent the results of three independent trials, and error bars represent one standard deviation above and below the mean. (**C**) The position of a laminar flow interface between the water from the pump and the water from the syringe was tracked to calculate the pressure generated by the pump. (**D**) The position of the laminar flow interface within the channel oscillates over time due to the cyclical application of force to the silicone tubing by the rotor.

This pump was designed for use in microfluidic applications to provide a low-cost tool that can be used for diagnostic purposes. One major application in microfluidic systems is control over fluid pressure within microfluidic channels. Applications of pressure driven flow include fluid based digital logic^10^, actuation of on-chip valves^17,29^, and control of laminar flow interfaces between fluids in a channel^1^. In order to test the ability of this pump to generate pressure for microfluidic applications, we used a Y-channel microfluidic device to track the position of a laminar flow interface over time. Controlling the position of a laminar flow interface between two co-flowing fluids in a single channel is a technique commonly used for applications such as controlling molecular diffusion^30^, patterning domains of small molecules within cells^31^, and for microfabrication within capillaries^32^. We tested the ability of our pump to generate stable laminar flow interfaces, and tested the dynamic pressure produced by the pump. The pump was attached to the inlet of one channel in a Y-channel PDMS microfluidic device, and a syringe suspended above the microfluidic device was attached to the other inlet (Fig. 3A). The pressure at the syringe inlet can be calculated based upon the height of the liquid in the syringe above the microfluidic device according the equation *P* = *ρgh*, where P is the pressure at the inlet, *ρ* is the density of the liquid, *g* is the acceleration due to gravity, and *h* is the height of the reservoir. An electric circuit analogy for microfluidic circuits was employed to calculate the pressure generated by the pump given the known pressure at the other inlet^33^. For fluid dynamics in low Reynolds number regimes such as those within a microfluidic channel, a simple relationship between flow rate and pressure can be derived from Hagen–Poiseuille’s law: $\Delta P=Q{R}_{H}$, where ΔP is the change in pressure over distance within a microfluidic channel, Q is the volumetric flow rate in the channel, and R_H_ is the hydraulic resistance of the channel, which can be calculated from the channel geometry. For a rectangular microfluidic channel where the height of the channel is much less than the width, R_H_ can be approximated by ${R}_{H}=12\eta L/w{h}^{3}$, where *η* = the viscosity of the fluid, *L* is the channel length, *w* is the channel width, and *h* is the channel height. The simplified equation relating pressure and flow rate in microfluidic chips is an analog to Ohm’s Law: $\Delta V=IR$, which is extensively used in electrical circuit analysis. Accordingly, the mathematics of circuit analysis, including Kirchhoff’s voltage and current laws, can be applied to microfluidic systems to determine pressures and flow rates within the system^33^. Change in pressure is analogous to change in voltage, volumetric flow rate is analogous to current, and hydraulic resistance is analogous to electrical resistance. Applying this analogy, an equivalent circuit model for the microfluidic device used in this experiment is presented in Fig. 3A. By observing the position of the laminar flow interface of two parallel fluid flows within the channel, it is possible to determine the relative flow rates of the two liquids according to the following relationship: $\frac{{w}_{1}}{{w}_{2}}=\frac{{Q}_{1}}{{Q}_{2}}$^33^. Employing this relationship in the circuit analysis of the system allows calculation of the unknown pressure generated by the pump, when compared to the known pressure due to gravity at the other inlet. The average pressure generated by the pump is a function of pump rotational speed (Fig. 3B). As the pump rotation speed is increased, the fraction of the flow in the microfluidic channel downstream of the Y-junction that is supplied by the pump increases, while the fraction of the flow supplied by the elevated fluid reservoir decreases (Fig. 3C). While the average pressure generated by the pump for a given rotational speed is constant, the dynamic pressure generated by the pump is not steady over time, but exhibits a cyclical fluctuation. As the rotor applies pressure to different parts of the tubing when turning, this generates a cyclical fluctuation in fluid pressure, which can be observed by tracking the position of the laminar flow interface in the channel over time (Fig. 3D). The dominant frequency of this oscillation can be calculated as angular frequency of the rotor multiplied by the number of rollers on the rotor (Supplementary Fig. S3).

### Programmable precision liquid handling

**Figure 4:**
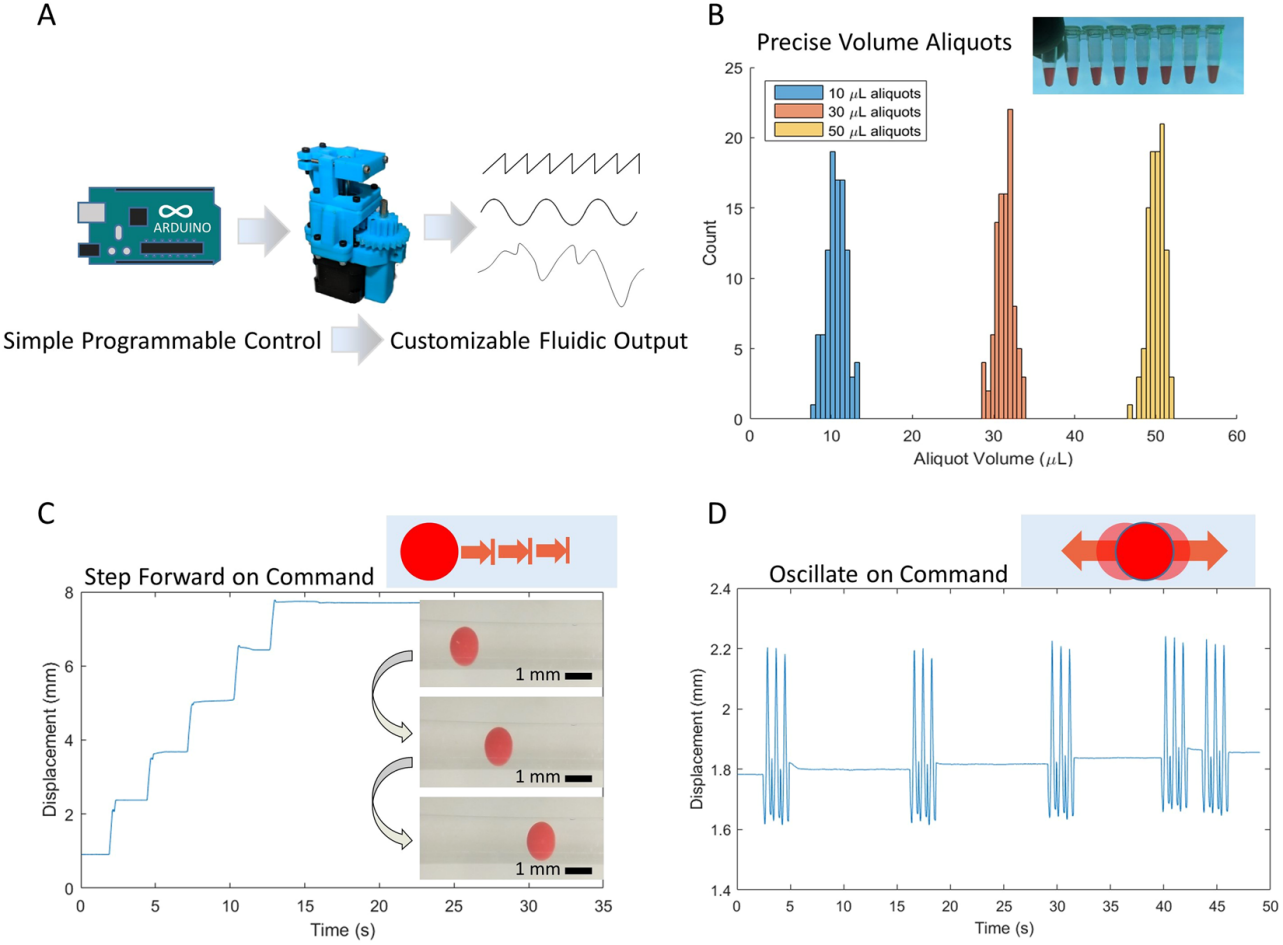
Programmable functions for custom liquid handling. (**A**) The pump is programmed with an Arduino microcontroller, which allows for user-defined arbitrary pumping behaviors. (**B**) The pump can be programmed to reliably and repeatedly dispense precise volumes of liquid. Histograms represents the volumes for 100 aliquots of deionized water at three different aliquot sizes. (**C**) The pump was programmed to drive a set volume of water through a 1 mm glass capillary under control of a user-operated switch. A cellulose acetate (CA) plastic sphere was placed in the capillary to aid in visualization of the moving fluid column, and the position of the sphere was tracked visually. (**D**) The pump was set to oscillate the column of water back and forth under user-defined control, and the movement of a CA sphere within the capillary was visually tracked.

As microfluidic systems expand into a growing number of application areas, the requirements for fluid pumping profiles will certainly expand as well. This pump was designed to be open-source and reprogrammable, in order to simply and rapidly modify the characteristics of the driven flow to meet operator-defined requirements (Fig. 4A). One application for which peristaltic pumps are commonly employed is liquid metering, so we tested the ability of our pump to precisely and repeatedly dispense aliquots of liquid. First, a calibration test was performed by clamping a 1.59 mm tube into the pump, and observing the flow rate by pumping deionized water onto a precision analytical balance at a constant rotor RPM. The resultant pump rate was used to calculate the time required to dispense a given volume of liquid. Tests were performed by pumping 100 aliquots each of 10 μL, 30 μL, and 50 μL onto the analytical balance. Additionally, 50 μL was individually dispensed into 8 PCR tubes to visually confirm aliquot volume (Fig. 4B).

Next, we expanded the versatility of our pump, the pump was reprogrammed to perform customized tasks under control of user input via rocker switches installed in the electronics box. Two custom programs were implemented: one to step forward, pumping fluid a specified amount before coming to rest (Fig. 4C), and one to oscillate the fluid back and forth (Fig. 4D). Example programs to run the pump are included in the Supplementary Materials (Supplementary Programs). To test these programs, the pump tubing was attached to a water filled glass capillary and a spherical cellulose acetate bead was placed in the capillary to visualize movement of the liquid column. The capillary was placed under a stereo microscope and the bead was observed with a camera as the operator toggled the switch on the electronics box to trigger the programmed response from the pump. The movement of the cellulose acetate bead within the channel was used to visualized the flow of liquid within the channel. Within the glass capillary, the low Reynolds number (≈1) means that viscous forces dominate over inertial forces, so the movement of the bead within the fluid closely tracks the movement of the column of fluid.

### Endothelial cell structural response under laminar fluid shear stress

**Figure 5:**
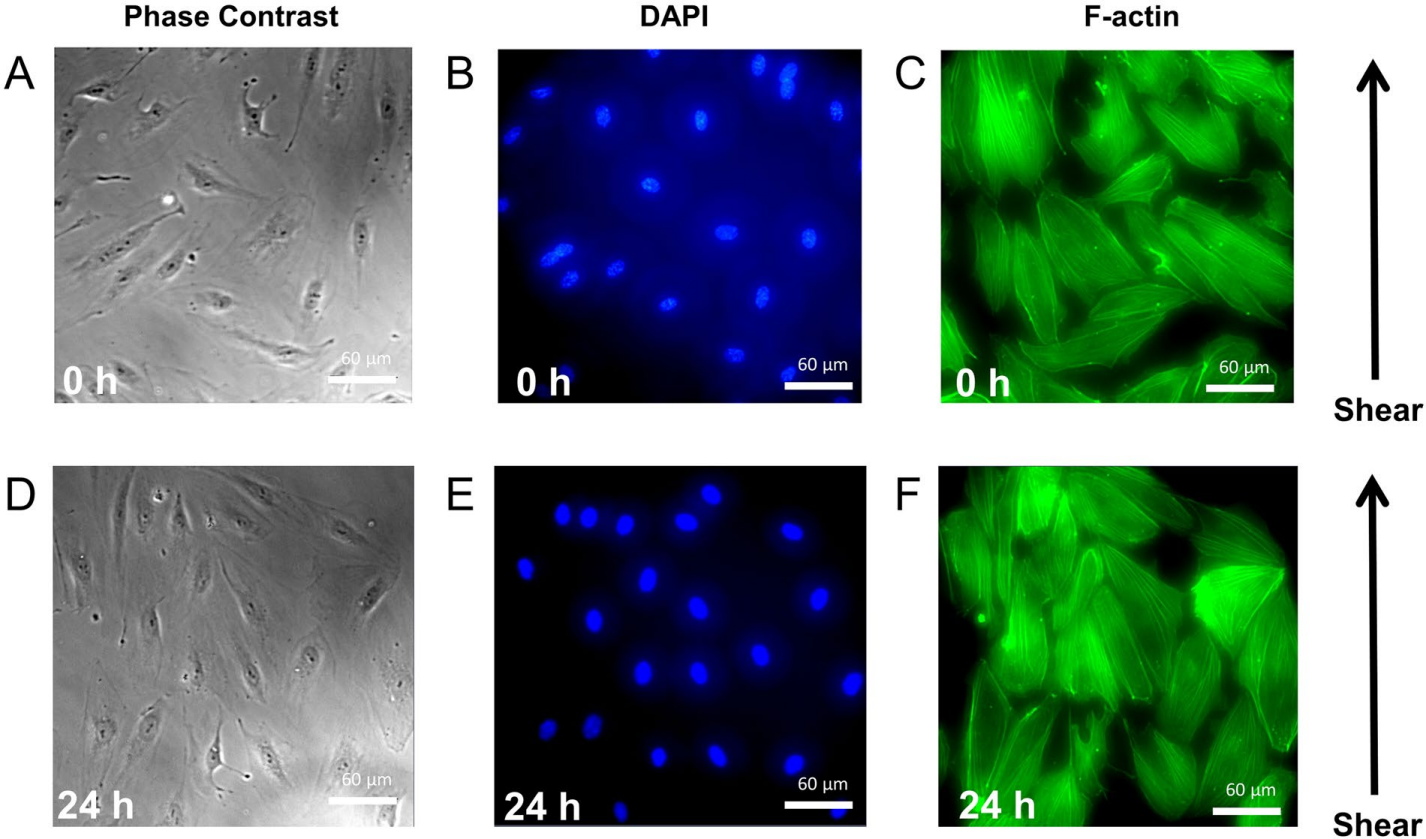
Laminar fluid shear stress causes alignment of endothelial cells. Cells in a monolayer within a microfluidic channel were exposed to shear stress from laminar flow generated by the pump, at 0.1498 Pa. Phase contrast and fluorescent images of Nucleus (DAPI) and F-actin at 0 h (**A**–**C**) and 24 h (**D**–**F**) of fluid shear stress.

We also tested the pumps ability to function as a tool for liquid handling in biological applications, we used the pump to generate laminar fluid shear stress on human vascular endothelial cells (HUVECs). Endothelial cells have been well documented to be responsive to fluid shear stress^34^. In fact, their alignment and change in morphology under laminar fluid shear stress is believed to protect the endothelium from many cardiovascular diseases and in general be barrier protective. For this study, we exposed a monolayer of HUVECs within a PDMS microfluidic channel to laminar fluid shear stress of 0.1498 Pa for 24 h. The endothelial cells exhibited a non-preferred orientation at the beginning of the experiment (Fig. 5A–C), while endothelial cells began to exhibit a more elongated shape and alignment along the direction of fluid shear at 24 h (Fig. 5D–F).

## Discussion

We have presented an open-source peristaltic pump built from 3D-printed plastic parts and common hardware, and demonstrated its applicability for small volume liquid handling in microfluidic applications, including generation of laminar flow interfaces and controlled sample movement under operator control. This pump represents a simple and inexpensive alternative to commercial pumping systems for microfluidics, with a number of key advantages. Unlike many of the comparable inexpensive peristaltic pumps on the market, this pump represents a complete liquid handling system, with its own power and control systems. The total price of the physical pump is approximately half the cost of the entire system, ≈$65, which is the number to compare to microfluidic pumps on the market with comparable performance specifications, such as the microfluidic peristaltic pump produced by Dolomite Microfluidics, which retails at $220 without power and control hardware, or the mp6 piezoelectric pumps produced by Bartels Mikrotechnik and sold through Servoflo, which costs $950 for a starter package. Although our pump is not the lowest cost option that has been developed for fluid handling in microfluidics (e.g., $2 reinforced latex balloons were developed by Thurgood *et al*. to provide a constant pressure source^21^), our design strikes a balance between reducing cost and enabling programmable flow profiles that are suitable for a wide range of applications. Furthermore, the open-source design allows for simple modifications to adapt the pump to application specific constraints such as tubing diameter, which affords this pump greater application flexibility in comparison to similarly priced commercial systems. Additionally, the 4:1 gearbox developed for this pump accepts attachments for a NEMA17 specified stepper motor, so it can be adopted for any other projects which require additional torque from a stepper motor. Programmability via the Arduino IDE allows for fine control over flow profiles, and integrated programmable switches in the electronics box can be easily programmed to control user-specified pumping routines, including stepping and oscillating flow. Fluids propelled by the pump are insulated from the hardware by the tubing, so sterility and contamination concerns are minimized in this system. Additionally, the peristaltic pump can be used to drive recirculating flow in a closed system, or to draw fluid from an arbitrarily large reservoir, which are key advantages over syringe pumps, one of the most common methods of microfluidic fluid control. Previously, our lab has presented a design for a 3D-printed feedback-controlled syringe pump that can act as a stable pressure source which may be better suited for applications which require precisely controlled pressure driven flow^1^. Together, these two pumps form a range of fluid handling technologies that are applicable to a wide range of point-of-care microfluidic applications.

We demonstrated the pump’s ability to be used for biological applications, and thus its feasibility as a point-of-care diagnostic system, by deploying our pump in conjunction with a microfabricated PDMS microchannel to exert a laminar fluid shear stress on a confluent HUVEC monolayer for 24 h. Our results revealed that (1) our pump can be used in conjunction with standard devices (such as a flow chamber) used in the biomedical field to study biological cells for a prolonged period of time, and (2) our pump was able to induce a laminar fluid shear stress-induced alignment of endothelial cells. Although, other groups have demonstrated the latter before, the uniqueness of this portion of our study may be found in the fact that the results we present here can be executed at a considerably lower operational cost and in the fact that this experiment utilizes materials that are much more accessible. We therefore believe our device will allow others who may be interested in running similar experiments to do so in previously inaccessible resource limited settings.

## Methods

### Design and construction of the peristaltic pump

All 3D-printed components were designed in 3D-computer aided design (CAD) software (Autodesk Inventor Professional 2016). The designs were exported as STL files and prepared for 3D-printing with Z-Suite (Z-SUITE Ver. 2.11.1.0, Zortrax), and parts were printed in Z-ABS plastic using a 3D-printer (Zortrax M200). The pump was assembled with a combination of commonly available hardware and 3D-printed components, using common tools. All 3D-printed parts are made available as STL files in the supplement, and a full bill of materials outlining the required hardware is also supplied in Supplementary Fig. S1. A detailed set of instructions for assembly is also included in the Supplementary Slide Deck, and an electrical schematic is included in Supplementary Fig. S4.

### Design and construction of microfluidic chips

Microfluidic devices were prepared by defining the channel geometry in 2D CAD software (Autodesk AutoCAD). These designs were then used as a mask for soft lithography. The CAD design was transferred to a maskless aligner (Heidelberg MLA100 Direct Write Lithographer), and lithographically patterned into SU-8 photoresist that was spin-coated onto a silicone wafer. Poly dimethlysiloxane (PDMS) (Sylgard 184, Dow Corning) was mixed at a 10:1 base to curing agent ratio, and poured over the wafer. The PDMS was degassed in a desiccator under vacuum pressure, and cured in an oven at 65 °C for 1 h. The PDMS was then removed from the wafer, and sliced into individual microfluidic devices. Inlet and outlet holes were punched in the devices using a blunt tipped stainless steel dispensing needle (Cat. No. 75165A675, McMaster Carr Supply Company). The devices were then oxidized in a plasma cleaner (PDC-32G, Harrick Plasma) for 30 s and plasma bonded to glass cover slips (22 × 40 mm). The devices were then incubated overnight at 65 °C to ensure a tight bond between the glass and the PDMS.

### Fluid flow rate determination

Flow rate was calculated by pumping deionized water into a weigh boat on an analytical balance (Adventurer AX, Ohaus) through silicone rubber tubing. Two sizes of tubing were tested: 1.59 mm OD tubing (Cat. No. 5236K204, McMaster Carr Supply Company), and 3 mm OD tubing (Cat. No. 5054K304, McMaster Carr Supply Company). Data was recorded via serial connection to a personal computer, and logged using SPDC Data Collection V2.03 (Ohaus). Weight measurements were recorded once per second. Data were analyzed in Matlab. The rotor rotation speed was measured by counting revolutions over a period of 2 min.

### Laminar flow interface in microfluidic channel

Silicone rubber 1.59 mm OD tubing (Cat. No. 5236K204, McMaster Carr Supply Company) was inserted into the pump. One end of the tubing was placed in a reservoir containing water supplemented with red food coloring (Red Food Color, McCormick Culinary), and the other end of the tubing was attached to one of the Y-inlets on the PDMS microfluidic device. The other inlet of the microfluidic device was connected to tubing running to a 10 mL syringe placed 2.47 meters above the microfluidic device, containing water and blue food coloring (Blue Food Color, McCormick Culinary). The outlet of the device was connected to tubing leading to an open waste collection container. Fluid was allowed to drain from the syringe through the device under gravitational power, while the speed of the pump was adjusted to vary the flow of fluid from the pump. The microfluidic device was placed onto the stage of a Nikon Eclipse Ts2 microscope, and viewed through a 4X objective lens. Video was captured with a camera in the eyepiece of the microscope (Celestron Digital Microscope Imager HD 5MP). The position of the laminar flow interface was calculated using ImageJ digital image processing software by binary thresholding, and tracking the position of the boundary of a binary object representing the flow.

### Precise volume aliquoting

The pump was prepared with 1.59 mm OD silicone rubber tubing (Cat. No. 5236K204, McMaster Carr Supply Company) and set to run at 30 RPM. Flow rate was calculated by pumping deionized water into a weigh boat on an Ohaus Adventurer analytical balance through silicone rubber tubing, and the flow rate was used to calculate the time required to aliquot a specified volume of water. The pump was then programmed to run 100 cycles for the calculated length of time, pausing for 5 s between cycles. Weight data was recorded via serial connection to a personal computer, and data was logged using SPDC Data Collection V2.03 (Ohaus). Weight measurements were recorded once per second. Data were analyzed in Matlab, and forward difference approximation was used to automatically identify intervals during which the pump was not running. The difference in weight between pauses was used to calculate the total amount of fluid pumped during the cycle. For pumping precise aliquots into individual PCR tubes, the pump was programmed to run for the specified interval when a switch was manually flipped. The dispensing end of the silicone tubing was placed in a new PCR tube for each aliquot.

### Visualization of operator controlled functions

The pump was programmed with the Arduino IDE to generate custom responses to an operator controlled switch. Fluid flow was visualized by placing a cellulose acetate 0.91 mm diameter red sphere (Cat. No. CAS-RED-1.3 0.91+/−0.05 mm-100, Cospheric) into a glass capillary with an inner diameter of 1 mm (Cat No. 13-678-20A, Fisher Scientific). The capillary was then connected to silicone rubber 1.59 mm OD tubing (Cat. No. 5236K204, McMaster Carr Supply Company), and water was pumped through the capillary. The capillary was placed under a stereo microscope (Carolina Biological Supply Company) at 2X magnification, and video was captured with a phone camera (Pixel 2, Google) viewing through the eyepiece. Movement of the cellulose bead was analyzed in ImageJ by converting the video to grayscale, thresholding to create a binary object, and tracking the coordinates of the centroid of the object over time. Small binary objects were filtered out to ensure that only a single object was tracked in each frame.

### Cell culture

Human umbilical vein endothelial cells (HUVECs) were cultured in medium 200 supplemented with large vessel endothelial supplement and 1% penicillin-streptomycin on 0.1% gelatin-coated flasks at 37 °C and 5% CO_2_.

### Fluid shear stress

The PDMS microchannel was coated with 0.1% collagen I and incubated at 37 °C for 4 h. After this time, Collagen I was flushed out of the chamber with PBS. HUVECs were subsequently seeded into the chamber, which was placed into a tissue culture incubator at 37 °C and 5% CO_2_ for at least 12 h before the experiment. Experimentation consisted of HUVECs being exposed to a fluid shear stress of 0.1498 Pa for 24 h in an incubator.

### DAPI-Phalloidin staining

HUVECs were first fixed by slowly pipetting 4% formaldehyde into the microchannel and then incubated at 37 °C for 15 min, followed by cell permeabilization with 0.2% Triton-X 100 for 5 min at 37 °C. Alexa Fluor 488 phalloidin (1:20) was next added to stain for f-actin for 2 h at 37 °C. After this time, fluoromount-G with DAPI was pipetted into the microchannel and HUVECs were then imaged using a Zeiss Inverted microscope with a 40x objective.

## Supplementary information

Supplementary Materials.

Dataset 1.

## Data Availability

All data available within the article or its Supplementary Materials.
