# Supplementary material for: Open-source, 3D-printed Peristaltic Pumps for Small Volume Point-of-Care Liquid Handling
Source: Sci Rep. 2020 Jan 31;10:1543. doi: 10.1038/s41598-020-58246-6 (PMC6994627; doi:10.1038/s41598-020-58246-6)
Supplement: Supplementary file 1 — Supplementary Materials. [file 41598_2020_58246_MOESM1_ESM.docx]

Open-source, 3D-printed Peristaltic Pumps for Small Volume Point-of-Care Liquid Handling

Supplementary Materials

**Authors:** Michael R. Behrens^1^, Haley C. Fuller^1^, Emily R. Swist^1^, Jingwen Wu^2^, Md. Mydul Islam^2^, Zhicheng Long^1^, Warren C. Ruder^1,^*, Robert Steward Jr.^2,^*

**Affiliation:**

^1^Department of Bioengineering, University of Pittsburgh, Pittsburgh, PA, United States

^2^Department of Mechanical and Aerospace Engineering, University of Central Florida, Orlando, FL, United States

***Corresponding Authors:**

Warren C. Ruder: [warrenr@pitt.edu](mailto:warrenr@pitt.edu)

Robert R. Steward: [rstewardjr@ucf.edu](file:///C:\Users\mrbeh\Documents\Grad%20School\Publications\Peristlatic%20Pump%20for%20Scientific%20Reports\Peristlatic%20Pump%20for%20Scientific%20Reports\Peristlatic%20Pump%20for%20Scientific%20Reports\rstewardjr@ucf.edu)

**Table of Contents**

**Supplementary Figures**

1. Bill of Materials
2. Individual Flow Rate Trials
3. Frequency of pressure oscillation
4. Electrical Schematic
5. Pressure vs Flow Rate
6. Power Efficiency

**Appendix A. How to assemble and use the peristaltic pump**

Embedded PDF

**Appendix B. Arduino Programs**

1. Run pump at constant speed
2. Oscillate
3. Step Forward

**Supplementary 3D Parts Files (included in Supplementary 3D STL Files.zip)**

1. Gear Box Base
2. Small Gear with Shelf
3. Stacked Gear
4. Gear Box Upper Case
5. Large Gear
6. Peristaltic Pump Stator (1.5 mm)
7. Peristaltic Pump Stator (3 mm)
8. Peristaltic Pump Lid (1.5 mm)
9. Peristaltic Pump Lid (3 mm)
10. Peristaltic Pump Clamp
11. Peristaltic Pump Rotor
12. Electronics Lid
13. Electronics Case

Figure S1. Bill of Materials.


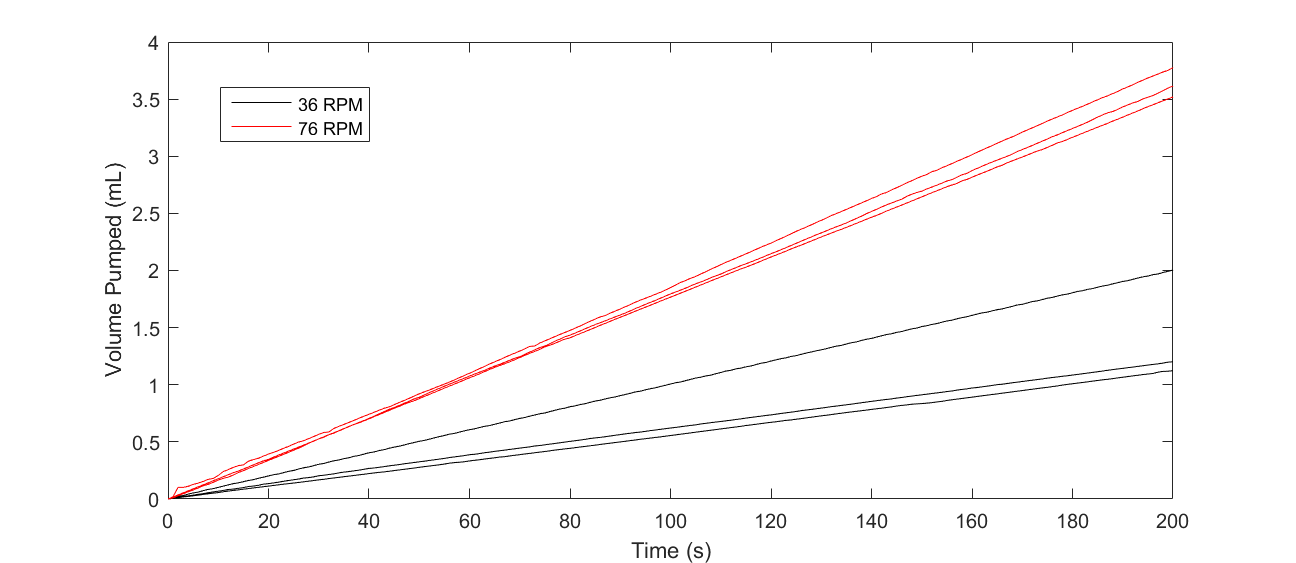


Figure S2. Individual Flow Rate Trials. Flow rate is constant for a constant RPM and tubing installation. The variation is introduced by varying the tension of the screws clamping the tubing into place within the pump, and by varying the tension of the tubing against the rotor. When the tubing is replaced the pump rate may not be exactly the same as it was previously. The three traces at each RPM here represent three separate tube installations in the pump.


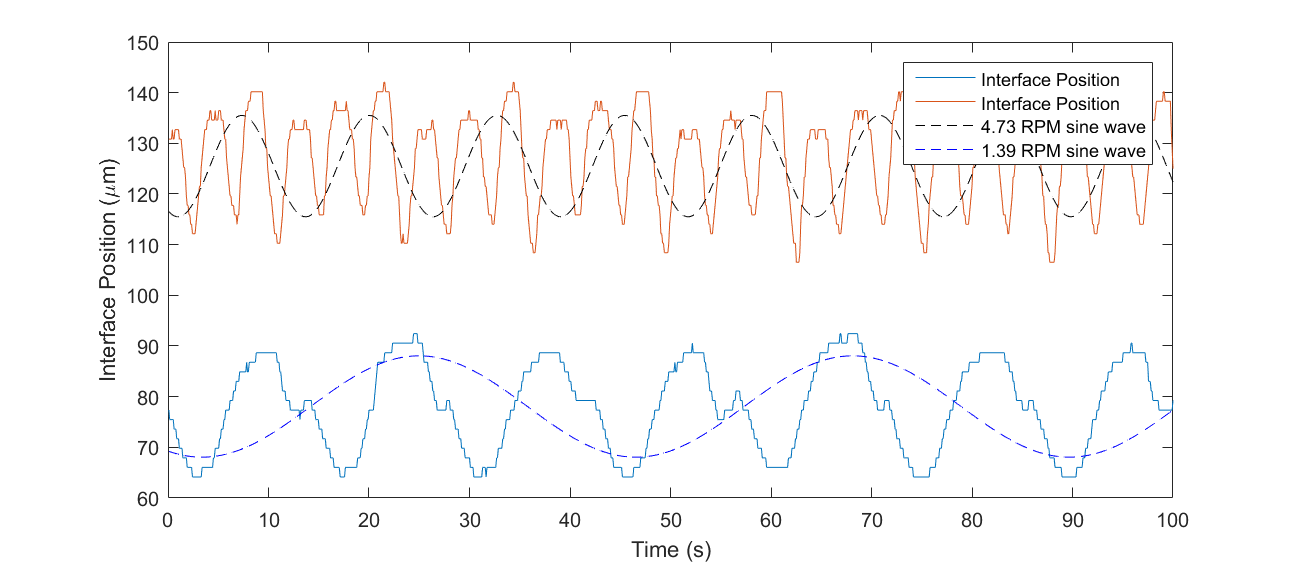


Figure S3. The frequency of pressure oscillations from the pump is a function of pump RPM. The frequency of oscillation of 3X the frequency of the pump, because of the three ball bearings on the rotor that apply pressure to the tubing.


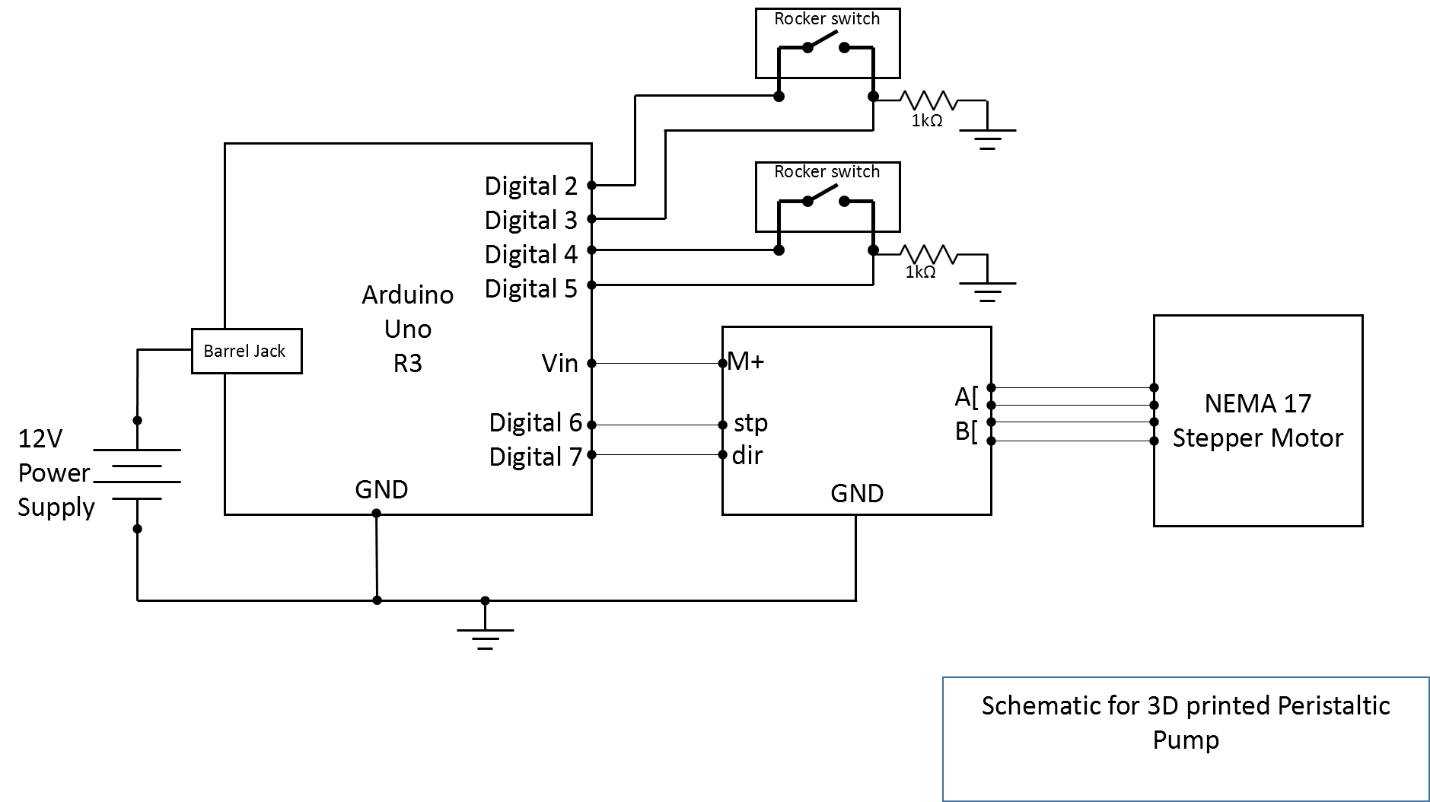


Figure S4. Electrical Schematic.


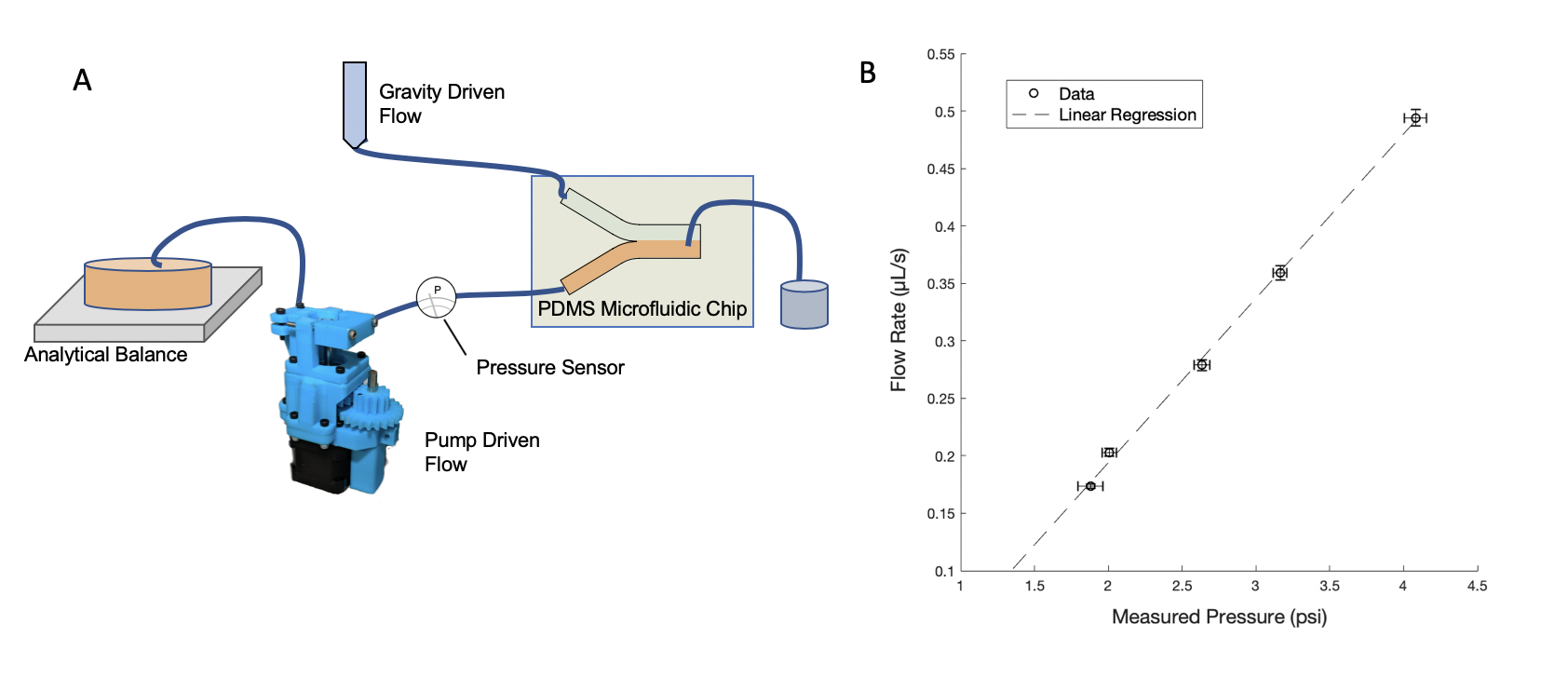


Figure S5. Pressure versus flow rate. A) Pressure drop across the microfluidic device was measured by recording the output from a pressure sensor placed in line between the pump and the microfluidic channel inlet. The flow rate from the pump was measured by drawing the fluid off of a reservoir upon an analytical balance (Ohaus Adventurer) and reading out the real time weight measurements with a computer. To obtain each measurement, the speed of the pump was set by programming the Arduino microcontroller, and pressure and flow rate were recorded over a time period of two minutes. The average values over two minutes were plotted. The experiment was repeated on three identical microfluidic devices. B) Measured pressure vs flow rate is shown. Error bars are representative of one standard deviation around the mean value taken at the same speed setting across three identical microfluidic devices.


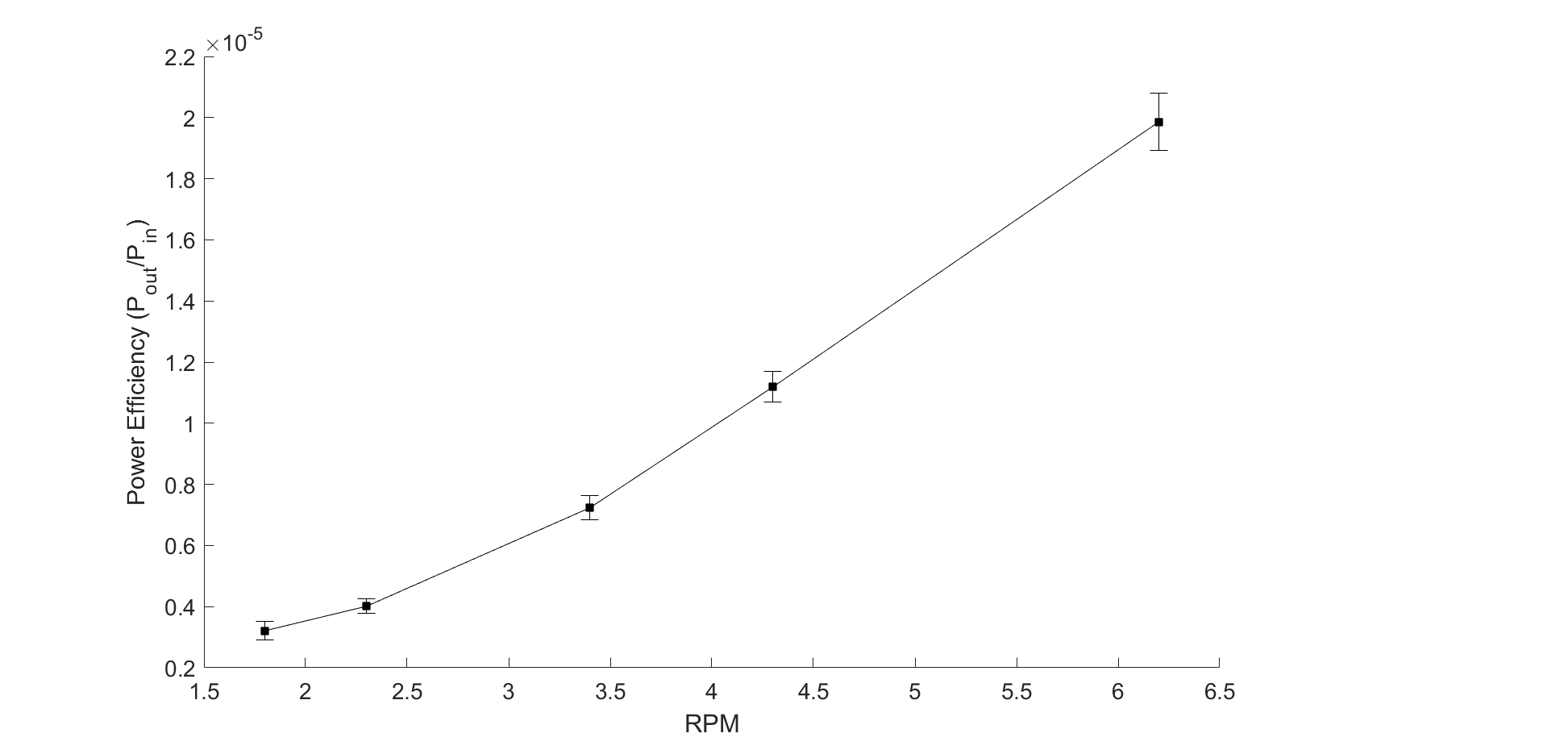


Figure S6. Power Efficiency. Pump efficiency as a function of pump rotations per minute is shown. Power consumption was determined by powering the pump from a laboratory power supply and multiplying the voltage by the current to obtain the electrical power drawn by the pump. The efficiency of the pump in converting electrical power to mechanical fluid power can also be calculated as P_out_ / P_in_ where P_out_ is calculated as output pressure multiplied by flow rate, and P_in_ is calculated as input voltage multiplied by input current. The input power was calculated to be 0.7 W, regardless of the RPM, while the output power was variable. Error bars represent one standard deviation.

**Appendix A. How to Assemble and Use the Peristaltic Pump. Embedded PDF.**

**Appendix B Arduino Code for the Pump.**

**Program 1: Run pump at constant speed**

/*

* this program is to control the peristaltic pump to run at a fixed speed, controlled by the frequency variable in the program.

*/

//Input the Frequency. Do not exceed 2. (is not actually in Hz. To verify RMP, the speed should be manually tested by counting revolutions)

float frequency = 1.8;

//**********************************************************************************************************************************************************************

float loopTime = (639.6/frequency-287.8);

float delayTime = loopTime/2;

unsigned long timeElapsed = 0;

int stp = 6;

int dir = 7;

int initialized = 0;

int power = 1;

int onSwitch = 0;

int sensor = 2;

int powerSwitch = 3;

int sensorRead = 0;

int directionSwitch = 5;

int dirSensor = 4;

int dirSensorRead = 0;

int previousDir = 0;

long previousMillis = 0;

long interval = 2000;

int a = 60;

// the setup function runs once when you press reset or power the board

void setup() {

// initialize digital pin 13 as an output.

pinMode(stp, OUTPUT);

pinMode(dir,OUTPUT);

pinMode(powerSwitch,OUTPUT);

pinMode(directionSwitch, OUTPUT);

pinMode(dirSensor,INPUT);

pinMode(sensor, INPUT);

pinMode(valve,OUTPUT);

digitalWrite(dir,HIGH);

digitalWrite(powerSwitch,HIGH);

digitalWrite(directionSwitch,HIGH);

Serial.begin(250000);

while (!Serial) {

;

}

}

// the loop function runs over and over again forever

void loop() {

if (onSwitch == 1)

{

if (initialized == 0)

{

for (int i = 500; i > 0; i--)

{

digitalWrite(stp, HIGH); // turn the LED on (HIGH is the voltage level)

delayMicroseconds(delayTime+i); // wait for a second

digitalWrite(stp, LOW); // turn the LED off by making the voltage LOW

delayMicroseconds(delayTime+i); // wait for a second

}

initialized = 1;

}

digitalWrite(stp, HIGH); // turn the LED on (HIGH is the voltage level)

delayMicroseconds(delayTime); // wait for a second

digitalWrite(stp, LOW); // turn the LED off by making the voltage LOW

delayMicroseconds(delayTime); // wait for a second

}

takeReading();

}

void takeReading()

{

sensorRead = digitalRead(sensor);

if(sensorRead == HIGH)

{

onSwitch = 1;

}

else

{

onSwitch = 0;

initialized = 0;

}

dirSensorRead = digitalRead(dirSensor);

if (dirSensorRead != previousDir)

{

initialized = 0;

}

previousDir = dirSensorRead;

if(dirSensorRead == HIGH)

{

digitalWrite(dir,HIGH);

}

else

{

digitalWrite(dir,LOW);

}

}

**Program 2: Oscillate**

/*

* this program is to control the peristlatic pump to oscillate fluids back and forth on command from a switch

*/

//Input the Frequency. Do not exceed 2. Note that this is not accurate in Hz, to verify the speed of the pump, manually check the RPM.

float frequency = 2; //2 to 1.66 to 1.33 to 1

//**********************************************************************************************************************************************************************

float loopTime = (639.6/frequency-287.8);

float delayTime = loopTime/2;

unsigned long timeElapsed = 0;

int stp = 6;

int dir = 7;

int initialized = 0;

int power = 1;

int onSwitch = 0;

int sensor = 3;

int powerSwitch = 2;

int sensorRead = 0;

int directionSwitch = 4;

int dirSensor = 5;

int dirSensorRead = 0;

int previousDir = 0;

int valve = 8;

long j = 0;

int valveState = 1;

long previousMillis = 0;

long interval = 2000;

int a = 60;

// the setup function runs once when you press reset or power the board

void setup() {

// initialize digital pin 13 as an output.

pinMode(stp, OUTPUT);

pinMode(dir,OUTPUT);

pinMode(powerSwitch,OUTPUT);

pinMode(directionSwitch, OUTPUT);

pinMode(dirSensor,INPUT);

pinMode(sensor, INPUT);

pinMode(valve,OUTPUT);

digitalWrite(dir,HIGH);

digitalWrite(powerSwitch,HIGH);

digitalWrite(directionSwitch,HIGH);

digitalWrite(valve,HIGH);

Serial.begin(250000);

while (!Serial) {

;

}

}

// the loop function runs over and over again forever

void loop() {

takeReading();

}

void takeReading()

{

sensorRead = digitalRead(sensor);

if(sensorRead == HIGH)

{

onSwitch = 1;

}

else

{

onSwitch = 0;

initialized = 0;

}

dirSensorRead = digitalRead(dirSensor);

if (dirSensorRead != previousDir)

{

initialized = 0;

}

previousDir = dirSensorRead;

if(dirSensorRead == HIGH)

{

//digitalWrite(dir,HIGH);

for (int j = 0; j<6; j++) //change direction 6 times

{

for (int i = 1000; i > 0; i--) //run 1000 steps in one direction

{

digitalWrite(stp, HIGH); // turn the LED on (HIGH is the voltage level)

delayMicroseconds(200); // wait for a second

digitalWrite(stp, LOW); // turn the LED off by making the voltage LOW

delayMicroseconds(200); // wait for a second

}

digitalWrite(dir,!digitalRead(dir));

}

while (dirSensorRead == HIGH)

{

dirSensorRead = digitalRead(dirSensor);

}

}

else

{

//digitalWrite(dir,LOW);

}

}

**Program 3. Step Forward**

/*

* this program is to control the peristaltic pump to step the fluid a set amount forward on command from a switch

*/

//Input the Frequency . Do not exceed 2.

float frequency = 1

//**********************************************************************************************************************************************************************

float loopTime = (639.6/frequency-287.8);

float delayTime = loopTime/2;

unsigned long timeElapsed = 0;

int stp = 6;

int dir = 7;

int initialized = 0;

int power = 1;

int onSwitch = 0;

int sensor = 2;

int powerSwitch = 3;

int sensorRead = 0;

int directionSwitch = 5;

int dirSensor = 4;

int dirSensorRead = 0;

int previousDir = 0;

int valve = 8;

long j = 0;

int valveState = 1;

long previousMillis = 0;

long interval = 2000;

int a = 60;

// the setup function runs once when you press reset or power the board

void setup() {

// initialize digital pin 13 as an output.

pinMode(stp, OUTPUT);

pinMode(dir,OUTPUT);

pinMode(powerSwitch,OUTPUT);

pinMode(directionSwitch, OUTPUT);

pinMode(dirSensor,INPUT);

pinMode(sensor, INPUT);

pinMode(valve,OUTPUT);

digitalWrite(dir,HIGH);

digitalWrite(powerSwitch,HIGH);

digitalWrite(directionSwitch,HIGH);

digitalWrite(valve,HIGH);

Serial.begin(250000);

while (!Serial) {

;

}

}

// the loop function runs over and over again forever

void loop() {

takeReading();

}

void takeReading()

{

sensorRead = digitalRead(sensor);

if(sensorRead == HIGH)

{

onSwitch = 1;

}

else

{

onSwitch = 0;

initialized = 0;

}

dirSensorRead = digitalRead(dirSensor);

if (dirSensorRead != previousDir)

{

initialized = 0;

}

previousDir = dirSensorRead;

if(dirSensorRead == HIGH)

{

//digitalWrite(dir,HIGH);

for (int i = 4000; i > 0; i--)

{

digitalWrite(stp, HIGH); // turn the LED on (HIGH is the voltage level)

delayMicroseconds(100); // wait for a second

digitalWrite(stp, LOW); // turn the LED off by making the voltage LOW

delayMicroseconds(100); // wait for a second

}

while (dirSensorRead == HIGH)

{

dirSensorRead = digitalRead(dirSensor);

}

}

else

{

//digitalWrite(dir,LOW);

}

}
